# Determining the effect of ocular chemical injuries on topical drug delivery

**DOI:** 10.1080/10717544.2021.1979124

**Published:** 2021-10-01

**Authors:** Ghazala Begum, Thomas Leigh, David Stanley, Ann Logan, Richard James Blanch

**Affiliations:** a Neuroscience and Ophthalmology, Institute of Inflammation and Ageing, University of Birmingham, Birmingham, UK; b NIHR Surgical Reconstruction and Microbiology Research Centre, University of Birmingham, Birmingham, UK; c School of Chemistry, University of Birmingham, Birmingham, UK; d Axolotl Consulting Ltd, Droitwich, UK; e Division of Biomedical Sciences, Warwick Medical School, University of Warwick, Coventry, UK; f Academic Department of Military Surgery and Trauma, Royal Centre for Defence Medicine, Birmingham, UK; g Department of Ophthalmology, University Hospitals Birmingham NHS Foundation Trust, Birmingham, UK

**Keywords:** Cornea, ocular chemical injury, drug delivery

## Abstract

Ocular chemical injuries (OCIs) commonly cause ocular damage and visual loss and treatment uses topical therapies to facilitate healing and limit complications. However, the impact of chemical injury on corneal barrier function and treatment penetration is unknown. Therefore, the aim of this study was to determine the effect of OCI on drug penetration and absorption. Porcine corneal explants were used to assess histological damage, electrical resistance, and the trans-corneal penetration/corneal adsorption of reference compounds (sodium fluorescein and rhodamine B) and dexamethasone. Corneal explants were injured with either 1 M sulfuric acid, or 1 M sodium hydroxide. Dexamethasone penetration was measured using high-performance liquid chromatography (HPLC) and that of fluorescein and rhodamine using fluorescence. Dexamethasone corneal adsorption was measured using enzyme-linked immunoabsorbant assay (ELISA). Both acid and alkaline injuries reduced trans-corneal electrical resistance. NaOH injury increased hydrophilic fluorescein penetration (NaOH 8.59 ± 1.50E–05 cm.min^−1^ vs. Hanks' Balanced Salt Solution (HBSS) 1.64 ± 1.01E–06 cm.min^−1^) with little impact on hydrophobic rhodamine B (1 M NaOH 6.55 ± 2.45E–04 cm.min^−1^ vs. HBSS 4.60 ± 0.972E–04 cm.min^−1^) and dexamethasone penetration (1 M NaOH 3.00 ± 0.853E–04 cm.min^−1^ vs. HBSS 2.69 ± 0.439E–04 cm.min^−1^). By contrast, H_2_SO_4_ decreased trans-corneal penetration of hydrophilic fluorescein (H_2_SO_4_ 1.16 ± 14.2E–07 cm.min^−1^) and of hydrophobic dexamethasone (H_2_SO_4_ 1.88 ± 0.646E–04 cm.min^−1^) and rhodamine B (H_2_SO_4_ 4.60 ± 1.42E–05 cm.min^−1^). Acid and alkaline OCI differentially disrupted the corneal epithelial barrier function. Acid injury reduced penetration of hydrophobic dexamethasone and rhodamine B as well as hydrophilic fluorescein, which may translate clinically into reduced drug penetration after OCI, while alkaline injury increased fluorescein penetration, with minimal effect on dexamethasone and rhodamine B penetration.

## Introduction

Ocular chemical injury (OCI) is an emergency requiring immediate action and treatment and can be caused by contact with either acid, such as sulfuric acid (battery acid), or alkali, such as calcium hydroxide (e.g. in cement) (Blanch et al., 2018). As acid and alkaline products are commonplace in the home and at work, OCI are common, comprising 11.5–22.1% of all ocular injuries (Wagoner, 1997; Sharma et al., 2018), with an annual incidence of 5.1–5.6 cases per 100,000 population in the USA and UK (White et al., 2015; Ghosh et al., 2019).

Contact with acid coagulates ocular surface tissue proteins, causing a barrier to further ocular penetration, so that weak acids cannot penetrate the biological tissue (Pfister, 1983; Singh et al., 2013). In comparison, alkali saponifies corneal cell membranes increasing permeability and alkali corneal penetration (Pfister, 1983; Brodovsky et al., 2000; Kuckelkorn et al., 2002), with pH change detectable in the aqueous humor of rabbits one minute after alkaline injury (Gérard et al., 1999).

Initial management of OCI is irrigation with large volumes of water or an inert buffer to dilute and remove the chemical and decrease further damage (Blanch et al., 2018). Topical formulations used to prevent infection, minimize scarring, and facilitate healing in the acute phase include antibiotics, collagenase inhibitors, and corticosteroids (Davis et al., 1997; Fish & Davidson, 2010; Baradaran-Rafii et al., 2017; Paschalis et al., 2017; Ramponi, 2017; Heng & Hamilton, 2018). These substances act on the cornea and penetrate the eye to mitigate intraocular damage. Corneal permeability to some of these substances, such as topical corticosteroids, has been studied in health (Nassr et al., 2009), but the effect of corneal injury on drug penetration into the eye has not been defined.

We therefore aimed to define the effect of acid and alkaline corneal injury on corneal absorption (modeling drug retention in the cornea) and permeability, defined as penetration across the cornea of dexamethasone and to reference these effects to polar/non-polar compounds in our *ex vivo* model of corneal penetration (Begum et al., 2020).

## Methods

### Corneal injury model

Corneal preparations were carried out as previously described (Begum et al., 2020). Briefly, porcine eyes were procured un-scalded and within two hours of death from Dissect Supplies (Birmingham, UK). Corneas were dissected from the globe and a 5 mm biopsy punch (Stiefel^®^, Brentford, UK and WellTech Rapid-Core, Taiwan, China) used to obtain approximately four corneal discs per cornea, which were each fitted separately into a CellCrown 96 well insert (Sigma-Aldrich, Gillingham, UK) with the epithelium facing up. The insert was placed in a black, clear F-bottom 96 well plate (Grenier Bio-one, Stonehouse, UK) that had been prefilled with 100 µL of Hanks’ Balanced Salt Solution (HBSS; Sigma-Aldrich, Gillingham, UK). Thirty microliters of HBSS was added over the corneal epithelium of control wells while 30 µL of 1 M sodium hydroxide (NaOH; Thermo Fisher, Waltham, MA) was added over the corneal epithelium to induce alkali injury or 30 µL of 1 M sulfuric acid (H_2_SO_4_; SLS, West Bridgford, UK) was added over the corneal epithelium to induce acid injury. Each solution was applied for 2 min before washing three times with HBSS. Neutral pH was confirmed using a pH strip before subsequent test solution application.

### Transcorneal epithelial resistance measurements

Wells of a Millicell 96-reciever plate (Millipore, Watford, UK) were filled with 200 μL HBSS. A 96-well culture plate (Millipore, Watford, UK) was placed on top of the receiver plate and the porcine corneal discs were placed epithelial side up in CellCrown 96 well inserts (Sigma-Aldrich, Gillingham, UK) which were then placed into 12 wells of the culture plate. The surface of the discs was then injured as follows: four with 20 μL of 1 M H_2_SO_4_, four with 20 μL of 1 M NaOH, and four were left uninjured (injury solutions washed off after 2 min as above). The same was added to 12 empty wells to serve as well-only tests. Corneal discs were covered with 50 μL HBSS and the transepithelial resistance recorded using the Millicell^®^ ERS-2 immediately after injury, and again at 30 min and 60 min after injury. Between measurements, the plate was incubated at 37 (±1)°C and 5% CO_2_. The process was repeated with *n* = 4 corneal sections from four different corneas for each injury condition, on two separate days.

### Hemotoxylin and eosin staining to assess corneal damage

After acid/alkali injury with NaOH and H_2_SO_4_ (as described above), corneal structure was histologically assessed using hemotoxylin and eosin (H&E) staining. Corneal inserts were opened, and cornea were gently removed and incubated in 4% paraformaldehyde (TAAB, Aldermaston, UK), then refrigerated overnight at 4 °C before replacing the PFA with 2 mL 15% sucrose (Sigma-Aldrich, Gillingham, UK) and leaving overnight. This last stage was repeated with a 30% sucrose solution. Cornea were embedded in OCT (Thermo Fisher, Waltham, MA) and the blocks frozen in dry ice. Corneal biopsy sections (15 μm) were taken from the corneal blocks using a cryostat (Model OT, Bright Instruments, Huntingdon, UK) onto Superfrost Plus^TM^ adhesion microscope slides (Thermo Fisher, Waltham, MA). Sections were stained by incubating in Harris hematoxylin (Sigma-Aldrich, Gillingham, UK) for 5 min before washing off with water and immersed in 1% acid alcohol, before being washed again and immersed in sodium bicarbonate solution (0.1% sodium bicarbonate; Thermo Fisher, Waltham, MA). Slides were then dehydrated with 95% ethanol (VWR, Poole, UK) before alcohol eosin Y staining (Sigma-Aldrich, Gillingham, UK) for one minute followed by rinsing with 70% ethanol and dehydration with ascending concentrations of ethanol. Finally, the slides were washed with Histo-clear (National Diagnostics, Nottingham, UK) and hard mounted with VectaMount Permanent Mounting Medium (Vector Laboratories, Peterborough, UK). Images were recorded using the eclipse TS100 microscope (Nikon, Minato City, Japan) at ×20 magnification.

### Fluorophore penetration and adsorption

Porcine corneal disks in inserts placed in wells of Millipore plates were injured with 1 M NaOH, 1 M H_2_SO_4_, or HBSS control as described above. After the injury and wash to neutral pH, 30 µL of 1% sodium fluorescein (Sigma-Aldrich, Gillingham, UK) and 50 µg.mL^−1^ rhodamine B (Sigma-Aldrich, Gillingham, UK) were applied to the corneal epithelial surface such that *n* = 3 for each application of HBSS-, 1 M NaOH-, and 1 M H_2_SO_4_-treated cornea. The Millipore plates were sealed with parafilm and incubated at 37(±1) °C and 5% CO_2_ for 60 min. The plate inserts with their cornea were carefully removed and the level of rhodamine B and fluorescein that had penetrated through the cornea into the underlying HBSS was measured in the wells of the black-welled plates using the FLUOstar^®^ Omega (BMG Labtech, Aylesbury, UK) and the Infinite^®^ M nano (Tecan, Reading, UK) microplate readers, respectively. For fluorescent analysis, the excitation and emission filters were set at; *λ*_exc_=485 nm, *λ*_emm_=520 nm for fluorescein, and *λ*_exc_=544 nm, *λ*_emm_=620 nm for rhodamine B, and absorbance measurements using the excitation wavelengths. Point values had the baseline of HBSS-only wells removed.

To qualitatively assess fluorophore adsorption into treated and control corneal tissue, inserts were opened, and corneas fixed in 4% paraformaldehyde overnight followed by 15% sucrose overnight and then a 30% sucrose overnight incubation all at 4 °C. Corneal disks were then embedded within OCT, frozen and sectioned on a cryostat. Sections (15 µm) were mounted with VectaSheild Antifade Mounting Medium containing 4′,6-diamidino-2-phenylindole (DAPI; Vector Laboratories, Peterborough, UK). The images were recorded on a Axioplan 2 imaging (Carl Zeiss, Cambridge, UK) microscope at ×20 magnification. The AxioCam HRc (Carl Zeiss, Cambridge, UK) was used to take the images in conjunction with Carl Zeiss^TM^ Axio Vision Rel 4.8 software (Carl Zeiss, Cambridge, UK) for multi-dimensional acquisition. Fluorescent filters were used to isolate DAPI, *λ*_exc_=377 ± 25 nm, *λ*_emm_=447 ± 30 nm; rhodamine B *λ*_exc_=560 ± 25 nm, *λ*_emm_=627.5 ± 27.5 nm, and fluorescein, *λ*_exc_=475 ± 17.5 nm, *λ*_emm_=530 ± 21.5 nm.

### HPLC measurement of dexamethasone adsorption to and penetration through injured corneas

Thirty microliters of 0.1 mg/mL^−1^ dexamethasone (Sigma-Aldrich, Gillingham, UK) were applied to 1 M NaOH-, 1 M H_2_SO_4_-, and HBSS-injured cornea discs as described above. After 60 min, the corneas were removed as described above and taken for dexamethasone adsorption analysis and the concentration in the remaining HBSS was used to assess dexamethasone penetration across the cornea. To determine dexamethasone adsorption into the corneal tissue, corneal discs were removed from the inserts, homogenized in 500 µL phosphate-buffered saline (PBS) and the homogenate was frozen in dry ice and stored at −80 °C. Later, 80 μL of ethyl acetate (Alfa Aesar, Thermo Fischer Scientific, Heysham, UK) was mixed into the thawed homogenate which was then micro-centrifuged on the IKA^®^ T-10 basic dispersing instrument (ULTRA-TURRAX^®^, IKA, Oxford, UK) at 4000 rpm for 10 min. Supernatant was mixed with an equal volume of 2 M NaOH, mixed and centrifuged for 1539×*g* (4000 rpm) for 10 min. Two hundred microliters of the upper layer was removed and placed in a fresh Eppendorf tube with 100 μL of sample resurrection buffer (1×). A 1:2 dilution was conducted in PBS, then a dexamethasone enzyme-linked immunoabsorbant assay (ELISA) (Cusabio, Houston, TX) was performed as detailed in the manufacturer’s protocol to assess adsorption into the corneal tissue.

To measure dexamethasone penetration through the cornea into the underlying HBSS, the HBSS samples were run through high-performance liquid chromatography (HPLC) using a Shimadzu detector (Shimadzu, Milton Keynes, UK), with an acetonitrile ammonium format gradient at an injection volume of 10 µL, measuring the absorbance at 239 nm (Chen et al., 2008). Dexamethasone concentration was calculated by measuring the area under the curve after the baseline of control HBSS had been removed. To calculate the molar concentration of dexamethasone in the wells, a calibration curve was first plotted for each experiment using dilutions of 100%, 10%, 5%, 1%, 0.5%, and 0.1% of the stock dexamethasone that had been added to the top of the cornea. These curves were created in duplicate.

### Statistical analysis

Data were collected and transferred to GraphPad Prism 8 for macOS (GraphPad Software, La Jolla, CA) for graphical presentation. SPSS Statistics version 24 (IBM Corp., Armonk, NY) was used for data analysis. Outliers more than three standard deviations from the mean were excluded. Generalized estimating equations were used to model penetration data to account for the repeated measures nature of the experimental design. Statistical significance was determined at *p*<.05. Unless otherwise specified, results are displayed as mean ± standard error of the mean (std. error).

## Results

### Disrupted corneal structure after chemical injury

**Figure 1.**
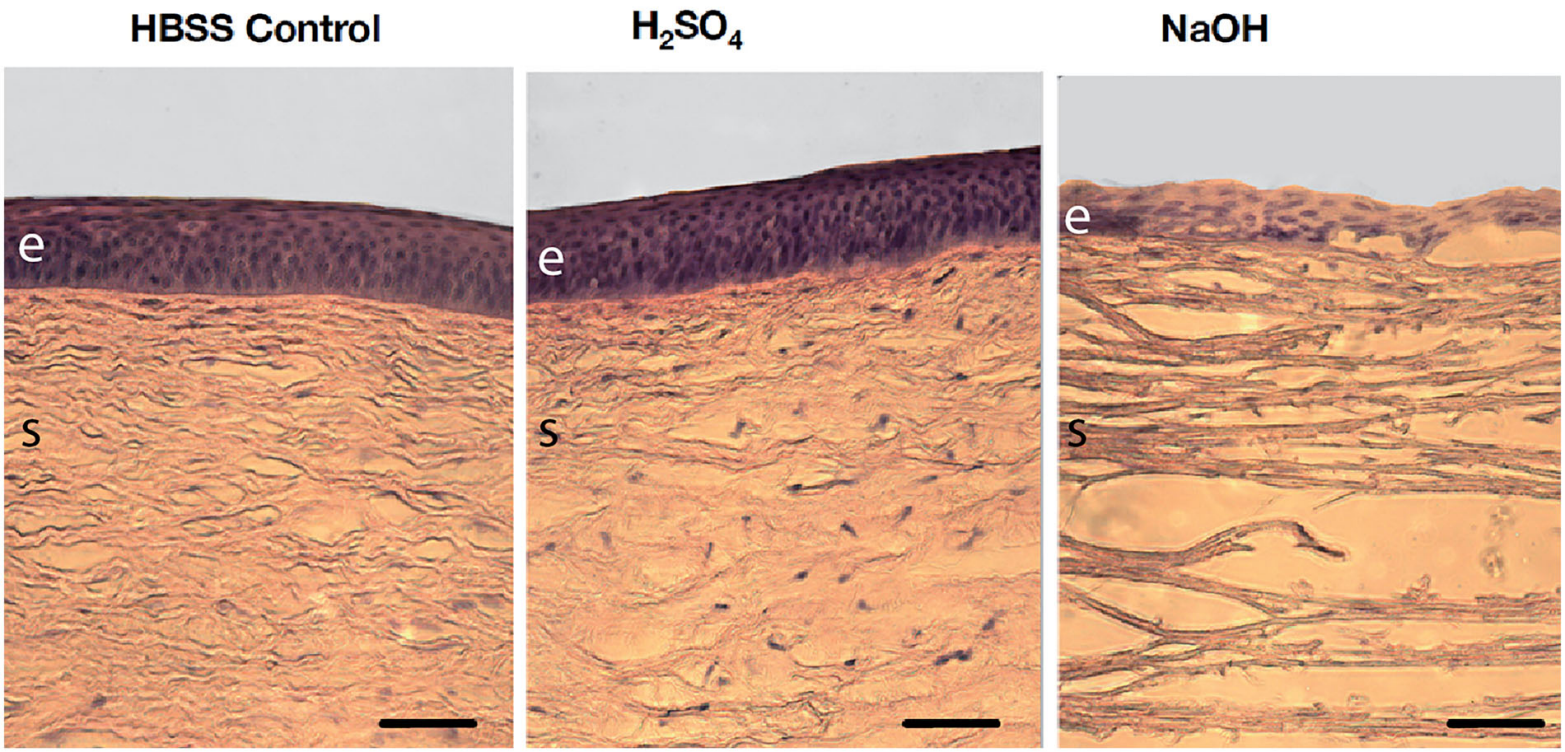
Altered structural integrity of the porcine corneal epithelium and stroma after chemical injury with 1 M H_2_SO_4_, or 1 M NaOH when compared to Hanks’ Balanced Salt Solution (HBSS) controls using hemotoxylin and eosin staining. Scale bar 100 µm; e: epithelium; s: stroma.

To identify changes in the porcine corneal structure after chemical injury with 1 M NaOH and 1 M H_2_SO_4_, injured cornea disks were compared to uninjured control HBSS corneal discs using H&E staining. Control cornea showed a clear intact epithelium and a well-preserved stroma (Figure 1). In comparison, the epithelium and stroma were relatively preserved after the application of acidic 1 M H_2_SO_4_ (Figure 1). The most extensive damage was observed after alkali treatment with 1 M NaOH, which led to extensive loss of the epithelial layer and profound stromal edema (Figure 1), with coagulation of collagen fibers into thinner layers.

### Decreased transcorneal epithelial electrical resistance after chemical injury

**Figure 2.**
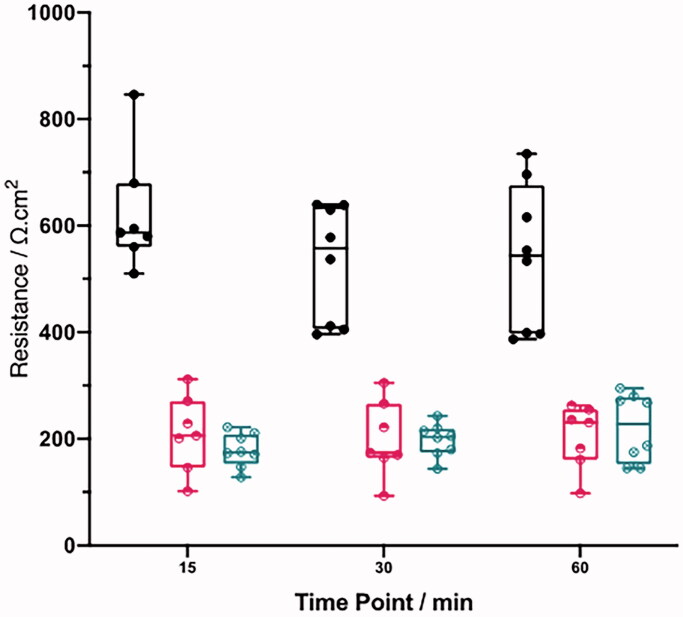
Reduced transepithelial electrical resistance (TEER) in porcine cornea injured with acid/alkali when compared to HBSS controls. Comparative transepithelial electrical resistance (TEER) of the cornea after a 2-minute exposure to HBSS (black), 1 M H_2_SO_4_ (red), or 1 M NaOH (blue) when measured immediately, at 30 min, and 60 min after the initial exposure. Data are represented as mean ± std. error where *n* = 8.

To determine the effect of acid/alkali injury on corneal barrier function, the transcorneal epithelial electrical resistance (TEER) was measured. Corneal disks were injured with 1 M H_2_SO_4_ and 1 M NaOH for 2 min before washing to neutral pH and compared to HBSS controls. TEER measurements were then taken immediately after pH normalization, at 30 min and 60 min. Over 60 min, the TEER levels remained consistent when HBSS was applied to the corneal surface (0 min, 623 ± 111; 30 min, 530 ± 109; 60 min, 540 ± 137). In comparison, acid and alkali injury both similarly decreased TEER (Figure 2), an effect that was detected immediately after injury (1 M H_2_SO_4_ 257 ± 149; 1 M NaOH 179 ± 32) and remained reduced for the 60 min (1 M H_2_SO_4_ 179 ± 32; 1 M NaOH 221 ± 64).

### Altered fluorophore adsorption in acid/alkali-injured cornea

**Figure 3.**
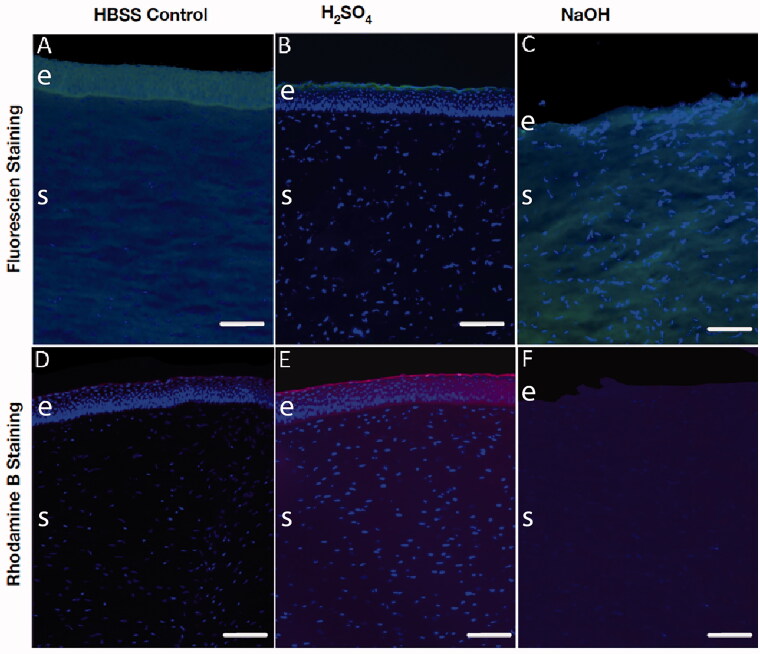
Fluorescent images demonstrating the altered adsorption of hydrophilic and hydrophobic agents after chemical injury. Porcine cornea injured with 1 M H_2_SO_4_ (B, E) and 1 M NaOH (C, F) were compared to HBSS controls (A, B) 60 min after the addition of fluorescein (A–C; hydrophilic, green signal) and rhodamine B (D–F; hydrophobic, red signal). Sections were counterstained with the nuclear dye DAPI. Scale bar = 100 µm; e: epithelium; s: stroma.

To assess the effects of chemical injury on compound adsorption and penetration, we initially assessed the adsorption into the cornea over 60 min of hydrophobic (fluorescein) and hydrophilic (rhodamine B) fluorescent dyes after injury by 1 M H_2_SO_4_ and 1 M NaOH and compared to HBSS controls. Fluorescent images showed clear fluorescein adsorption throughout the corneal epithelium and the stroma in HBSS control corneal discs (Figure 3(A)). After 1 M H_2_SO_4_ treatment, fluorescein remained on the epithelial surface and did not show adsorption into the epithelium layers or the stroma (Figure 3(B)). By contrast, after 1 M NaOH injury which stripped the epithelium, fluorescein was observed throughout the stroma (Figure 3(C)). When rhodamine B was applied, the HBSS treated cornea did not show any adsorption into either the epithelial layers or the stroma (Figure 3(D)). In the 1 M NaOH (Figure 3(E)) injured cornea, there was some stromal adsorption of rhodamine and clear rhodamine B adsorption to the epithelium and stroma after 1 M H_2_SO_4_ injury (Figure 3(F)).

### Penetration of hydrophilic and hydrophobic dyes through the cornea is disrupted by chemical injury

**Figure 4.**
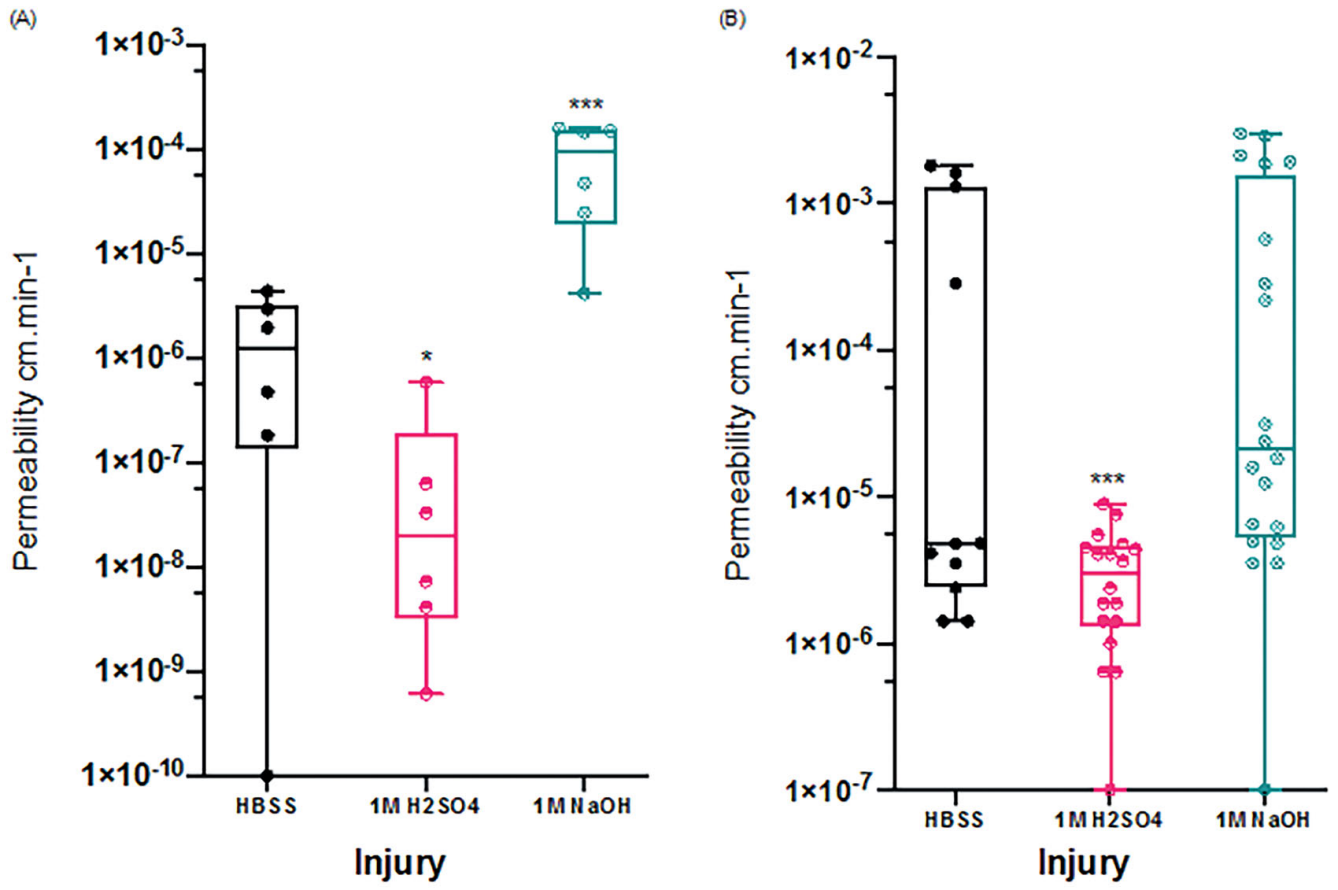
Altered rate of penetration of fluorescein (A) and rhodamine B (B) across the cornea after HBSS (black), 1 M H_2_SO_4_ (red), or 1 M NaOH (blue) application to the corneal surface. Data represent *n* = 3 experiments where *n* = 5 cornea in each treatment group. Data are presented as mean ± std. error where ***p<.001 and *p<.05.

To investigate if chemical injury altered the penetration of hydrophobic and hydrophilic agents through the cornea, we measured fluorescein and rhodamine B accumulation in the HBSS underlying the cornea and calculated a rate of transport. During the 60 min of dye application to the upper corneal surface, the permeability of intact (HBSS applied) cornea to fluorescein was 1.64 ± 1.01E–06 cm.min^−1^ (Figure 4(A)). Acid damage by 1 M H_2_SO_4_ reduced the dye penetration rate to 1.16 ± 14.2E–07 cm.min^−1^ (Figure 4(A); *p*=.012), which represents 7% of the mean of the uninjured cornea. There was strong evidence that alkali damage increased the rate of fluorescein corneal penetration to 8.59 ± 1.51E–05 cm.min^−1^ (Figure 4(A); *p*<.001), which is an increase of 5358% from the mean of the uninjured cornea.

In comparison to the fluorescent images that showed rhodamine B was not as well adsorbed to corneal tissue after HBSS and 1 M NaOH exposure, there was no change in the rate of penetration of rhodamine B across the corneal tissue after alkaline injury (Figure 4(B); HBSS 4.60 ± 0.972E–04 cm.min^−1^, 1 M NaOH 6.55 ± 2.45E–04 cm.min^−1^, *p*=.386). In contrast, there was a strong reduction in the rate of rhodamine B penetration across cornea damaged by 1 M H_2_SO_4_ to 4.60 ± 1.42E–05 cm/min (Figure 4(B); *p*<.001), which represents a 90% reduction from the mean of the uninjured (HBSS) cornea.

### Corneal penetration and adsorption of dexamethasone

**Figure 5.**
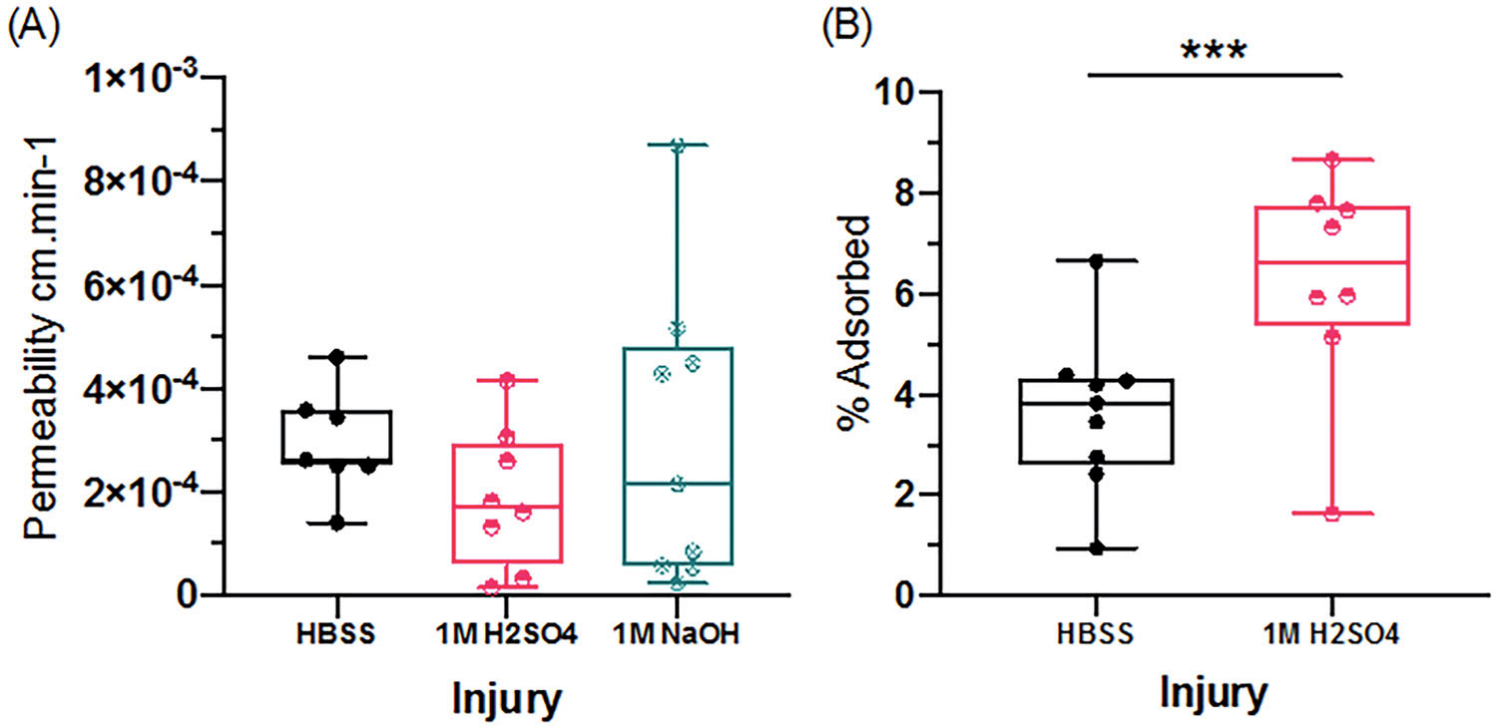
Dexamethasone penetration and adsorption analysis. (A) Dexamethasone penetration measured using high-performance liquid chromatography (HPLC) after 60 min. (B) Dexamethasone corneal adsorption measured by ELISA after H_2_SO_4_ injury. Data are representative of the mean ± std. error where each group consists of *n* = 5 cornea where ***p<.001.

To assess a potential clinical impact of OCI on penetration of a standard ocular medication, we tested the rate of penetration of dexamethasone through the injured cornea. After dexamethasone application, permeability through the intact (HBSS) cornea was 2.69E–04 cm.min^−1^ (Figure 5(A); std. error 4.39E–05; 95% CI 1.83–3.55). There was weak evidence that acid damage by H_2_SO_4_ reduced the rate of corneal permeability to dexamethasone to 1.88E–04 cm.min^−1^ (Figure 5(A); std. error 6.46E–05; 95% CI −17.3 to 1.19; *p*=.088), which represents a decrease of 30% from the mean of the HBSS cornea. There was no evidence that alkali damage affected the rate of corneal penetration (Figure 5(A); 3.00E–04, std. error 8.53E–05, 95% CI for the difference from HBSS: 1.12–1.74E–06 cm.min^−1^; *p*=.674).

As acid injury reduced corneal dexamethasone penetration, we examined the effect of H_2_SO_4_ acid injury on corneal dexamethasone absorbance, by measuring the concentration of dexamethasone in homogenized cornea using ELISA. Intact (HBSS) cornea adsorbed 3.50% of applied dexamethasone (std. error 0.47; 95% CI 2.57–4.42). Acid injury increased adsorption of dexamethasone into the cornea by 2.29% (Figure 5(B); std. error 0.638; 95% CI 3.54–1.04%; *p*<.001).

## Discussion

OCI requires immediate intervention and careful management. However, the impact of ocular surface damage on the penetration and adsorption of topical therapeutics is not previously defined. We induced chemical injury in porcine corneal discs with sulfuric acid and sodium hydroxide and found significant impairments in corneal barrier function with altered adsorption into and penetration across tissue of hydrophobic and hydrophilic agents, which were differentially obstructed by alkali and acid injuries. The penetration of hydrophobic rhodamine B and dexamethasone was reduced by acid injury and corneal adsorption (drug retention) increased, which has implications for the use of hydrophobic topical therapies as part of OCI treatment strategies.

OCI was replicated in our model by topically applying either sulfuric acid or sodium hydroxide. These chemicals can be found in battery acid and industrial cleaners representing some of the most common injuries. Both alkali and acid injury significantly altered corneal barrier integrity as measured by TEER, consistent with previous reports (Guimera et al., 2012; Fukuda & Sasaki 2016; Uematsu et al. 2016; Kaluzhny et al., 2018; Begum et al., 2020). Microscopy of alkali-injured corneas demonstrated significant structural abnormalities of the epithelium and stroma and the drop in TEER most likely reflects immediate epithelial damage after acid or alkaline injury. The damage induced in the alkali injured cornea was severe, which can be explained by the saponification of the lipid bilayers causing a breakdown of both the epithelial and stromal structural integrity, with consequent major perturbation of the barriers to tissue penetration. After alkali injury, the greatest change in permeability seen was to the hydrophobic compound, fluorescein (log *P* 3.92) (Kaler et al., 2007). However, the same effect was not seen for penetration of the hydrophilic agents rhodamine B (log *P* 1.95) (Kaler et al., 2007) or dexamethasone (log *P* 1.83) (Lipinski et al., 1997; Lombardo et al., 2000), that showed similar to levels of penetration to those observed in uninjured (HBSS) controls. This suggests that after alkali injury, the penetration of polar/non-polar agents is differentially affected and that of hydrophilic agents is not dependent on the structural integrity of the epithelial lipid membranes.

While similarly affecting TEER, structurally, acidic damage did not disrupt the corneal epithelium to the same extent as alkaline damage. In this case, the superficial protein coagulation induced may provide a physical barrier to compound penetration. Consequently, the hydrophobic fluorescein adsorbed into the corneal epithelial surface where the protein coagulation is presumed to occur, suggesting retention and build-up here rather than penetration across the corneal tissue. Absorption of the hydrophilic rhodamine B was also comparably reduced, with a build up at the epithelial surface and reduced penetration across the cornea. Accordingly, penetration of hydrophilic dexamethasone was reduced, while corneal adsorption was increased. Acid-injury therefore reduces ocular penetration of both hydrophilic and hydrophobic compounds.

We acknowledge the limitations of this study, including the static nature of the assay without effects of blinking, the lack of active inflammatory processes in response to injury (although significant immune cell infiltration would not be expected within the first 60 minutes after injury) and a lack of physiologic tear film and ocular surface proteins, which may exert a buffering function. All three chemicals studied have a similar relatively low molecular weight, limiting extrapolation of the results to large molecular weight drugs, such as potential monoclonal antibody therapies.

In conclusion, acid OCI increased dexamethasone and rhodamine B corneal adsorption (retention) and reduced their penetration across the cornea. By contrast, while the severe compromise of the epithelial layer after alkali OCI did not affect corneal dexamethasone penetration, it did increase penetration of the hydrophilic compound sodium fluorescein.
